# FAM172A inhibits EMT in pancreatic cancer via ERK-MAPK signaling

**DOI:** 10.1242/bio.048462

**Published:** 2020-02-07

**Authors:** Ying Chen, Peihui Liu, Di Shen, Han Liu, Lepeng Xu, Jian Wang, Daguang Shen, He Sun, Hongkui Wu

**Affiliations:** Department of Intervention Therapy and Vascular Surgery, The Central Hospital of Huludao City, Huludao City, Liaoning Province, 125399 China

**Keywords:** FAM172A, EMT, Pancreatic cancer, ERK-MAPK

## Abstract

FAM172A, as a newly discovered gene, is little known in cancer development, especially in pancreatic cancer (PC). We investigated the potential role and molecular mechanism of FAM172A in epithelial to mesenchymal transition (EMT) in both human clinical samples and PC cells. FAM172A was downregulated in human PC tissues compared with that in non-cancerous pancreas cells by immunohistochemistry and qRT-PCR. FAM172A expression was negatively associated with tumor size (*P*=0.015), T stage (*P*=0.006), lymph node metastasis (*P*=0.028) and the worst prognosis of PC patients (*P*=0.004). Meanwhile, a positive relationship between FAM172A and E-cadherin (E-cad) (r=0.381, *P*=0.002) was observed in clinical samples, which contributed to the better prognosis of PC patients (*P*=0.014). FAM172A silencing induced EMT in both AsPC-1 and BxPC-3 cells, including inducing the increase of Vimentin, MMP9 and pERK and the decrease of E-cad and β-catenin expression, stimulating EMT-like cell morphology and enhancing cell invasion and migration in PC cells. However, MEK1 inhibitor PD98059 reversed FAM172A silencing-enhanced EMT in PC cells. We conclude that FAM172A inhibits EMT of PC cells via ERK-MAPK signaling.

## Abstract (precis)

**Summary:** FAM172A inhibits EMT in pancreatic cancer via specifically regulating ERK-MAPK signaling.

## INTRODUCTION

Pancreatic cancer (PC), as a deadly malignancy, ranks as the fourth leading cancer-related cause of death in Europe (Rawla et al., 2019). It is also ranked as the top first incidence, and second age-standardized cause of mortality in Chinese males (Chen et al., 2016). The poor prognosis for PC patients is mainly due to its aggressive biological behavior, which is boosted by epithelial-to-mesenchymal transition (EMT). EMT, as one of the most distinctive and critical features in cancer (Gaianigo et al., 2017), promotes the loss of epithelial character and the gain of invasive mesenchymal properties, and finally contributes to the highly malignant phenotype in PC (Wang et al., 2017).

Family with sequence similarity 172 member A (FAM172A), cloned from human aortic tissues, is subsequently observed in human endothelial and vascular smooth muscle cells, and macrophages (Li et al., 2010). However, its biological function in disease states is still not clear. As the imbalance of the cell cycle and apoptosis is closely related to the key characteristics of malignant tumors, FAM172A dysregulation is a potential contributing factor in cell transformation (Feng et al., 2013). Recently, FAM172A has been reported to take part in the development of several cancers, such as human liver, colorectal and papillary thyroid cancers (Qian et al., 2016; Feng et al., 2013; Li et al., 2016). However, its definite role (oncogene or tumor suppressor) in cancers remains controversial and the corresponding molecular regulation is poorly elucidated. In the last few decades, an epidemic of diabetes and obesity has been spreading worldwide, contributing to an increase in incidence of PC (Paternoster and Falasca, 2020). FAM172A was upregulated by high glucose in a concentration- and time-dependent manner (Li et al., 2010), which indicated that FAM172A may be involved in the pathogenesis of diabetic related diseases. It is well known that PC is a multi-stepped progression involving a series of genetic and epigenetic events leading to the transformation of the normal ductal cells to carcinoma. Therefore, we intend to investigate the potential role of FAM172A in PC development, which has not been reported, to our knowledge.

The ERK-MAPK signaling plays a significant role in regulating cell proliferation, differentiation, survival, migration senescence and apoptosis via transmitting signals from cell surface receptors (Sun et al., 2015). ERK-MAPK signaling acts as an oncogene in various solid tumors, such as glioma, prostate, thyroid, ovarian and non-small cell lung cancers (Montagut and Settleman, 2009; Sun et al., 2015). Here, we first found that FAM172A inhibits EMT in PC via ERK-MAPK signaling, which supplies a new gene target therapy for PC patients.

## RESULTS

### The clinicopathological significance of FAM172A expression in PC tissues

**Fig. 1.**
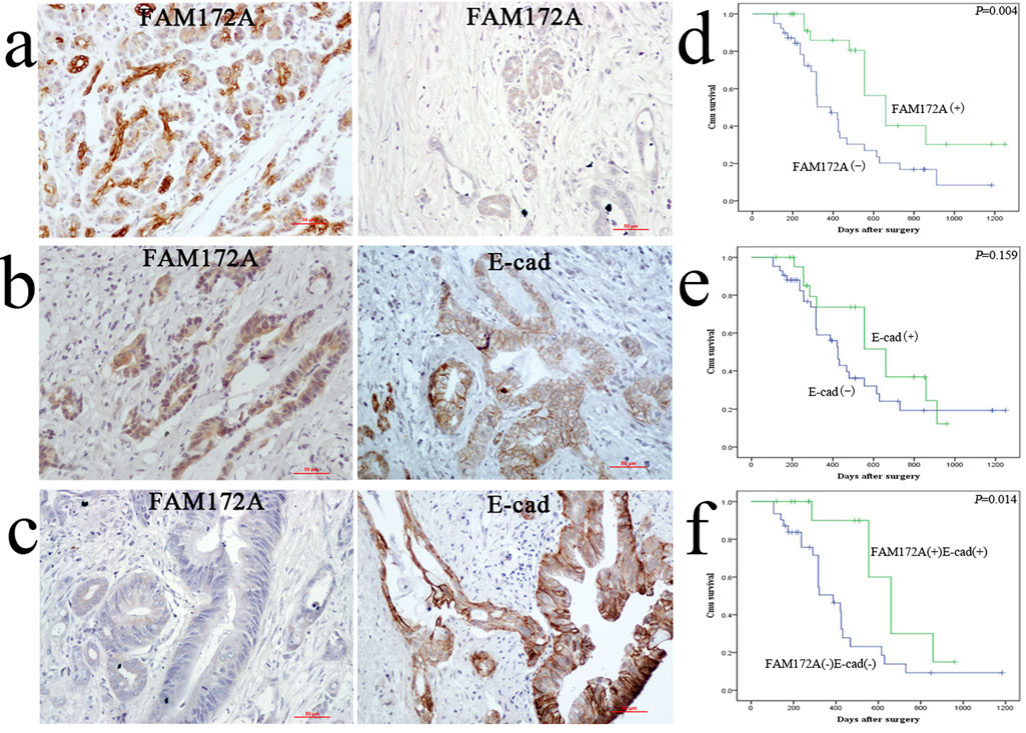
**FAM172A and E-cad expression in PC tissues by IHC and their expression with the survival of PC patients.** (A) FAM172A expression in PC (right side) and paired adjacent pancreas (left side). Scale bars: 50 µm. (B) FAM172A positive expression and E-cad normal expression in one case of PC tissues. Scale bars: 50 µm. (C) FAM172A negative expression and E-cad abnormal expression (cytoplasm expression) in another one case of PC tissues (×100 magnification). (D) Positive (+) and negative (−) expression of FAM172A was plotted against overall survival time. (E) Normal (+) and abnormal (−) expression of E-cad was plotted against overall survival time. (F) Cooperative expression of these two proteins was plotted against overall survival time.

FAM172A was mainly expressed in cytoplasm in both PC and paired pancreas. As described previously (Sheng et al., 2017b), E-cad membrane expression in PC was identified as normal expression (Fig. 1B), while E-cad negative and cytoplasmic expression were considered as abnormal expression (Fig. 1C). Immunohistochemistry (IHC) showed FAM172A positive expression in PC samples was much lower than that in paired adjacent normal pancreas cells (26/65, 40% versus 48/65, 73.8%, *P*<0.01) (Fig. 1A). In most serial sections, PC tissues with FAM172A positive expression was associated with E-cad normal expression (Fig. 1B), and vice versa (Fig. 1C). Spearman’s correlation test confirmed a positive relationship between FAM172A and E-cad in 65 PC samples (r=0.381, *P*=0.002) (Table 1).

**Table 1.**
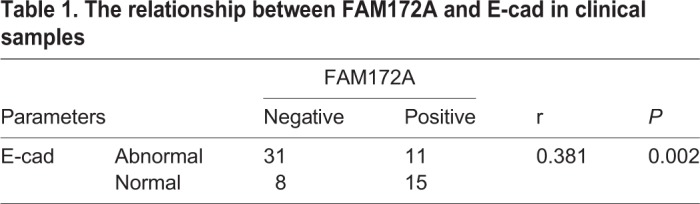
**The relationship between FAM172A and E-cad in clinical samples**

Chi-squared test showed that FAM172A was negatively associated with tumor size (*P*=0.015), T stage (*P*=0.006), lymph node metastasis (*P*=0.028), but had no relationship with age, gender, tumor location, differentiation, vascular invasion and preoperative CA199 level in PC patients (*P*>0.05) (Table 2). Patients with positive FAM172A expression had a much better overall survival compared with patients with FAM172A negative expression (*P*=0.003) (Fig. 1D). Though E-cad had no association with the prognosis (*P*=0.159) (Fig. 1E), patients with the cooperative expression of FAM172A and E-cad contributed to the better overall survival of PC patients (*P*=0.014) (Fig. 1F). In multivariate model, FAM172A was an independent favorable prognostic indicator (*P*=0.020) (Table 3).

**Table 2.**
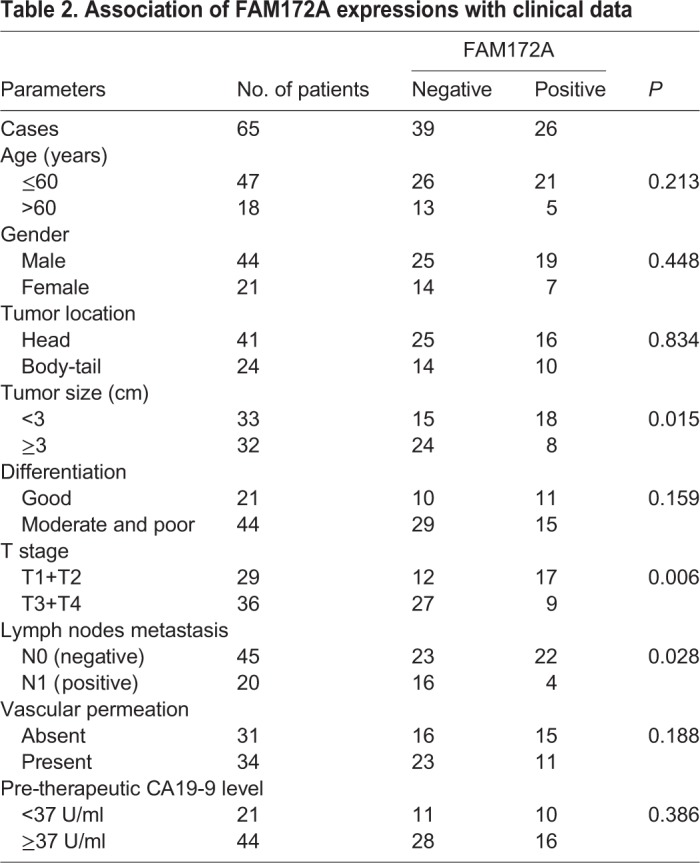
**Association of FAM172A expressions with clinical data**

**Table 3.**
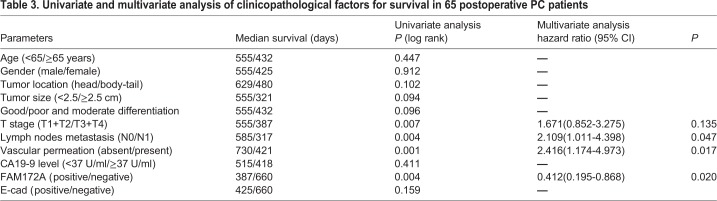
**Univariate and multivariate analysis of clinicopathological factors for survival in 65 postoperative PC patients**

**Fig. 2.**
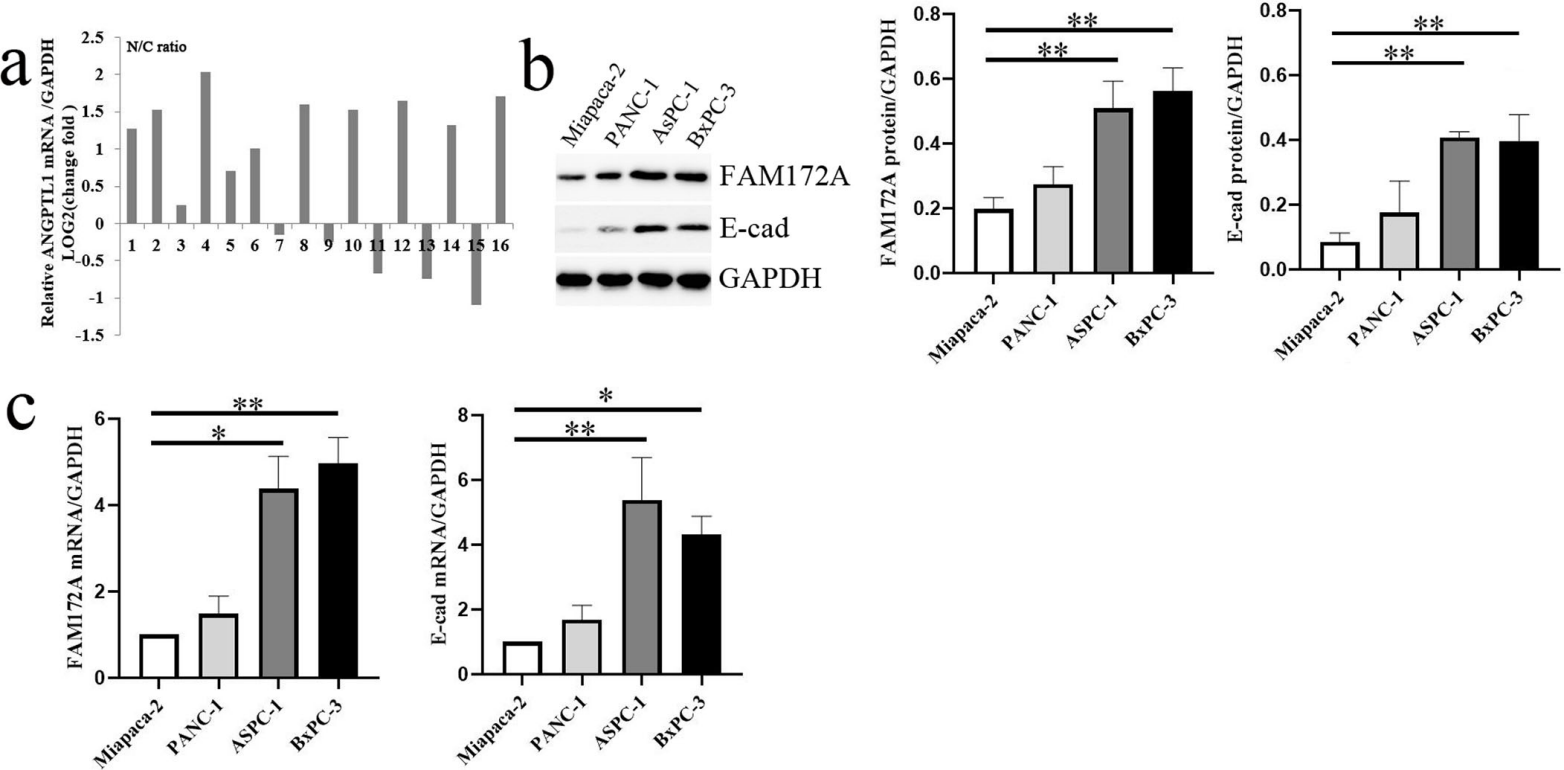
**FAM172A and E-cad expression in PC tissues and cell lines.** (A) FAM172A mRNA expression in 16 PC and paired normal pancreas by qRT-PCR (N/C ratio). (B,C) FAM172A and E-cad protein (B) and mRNA (C) expression in PC cell lines. C, PC tissues; N, paired normal pancreas. Bars indicate±s.e. *P<0.05, **P<0.01 compared with the control.

Consistent with the IHC results, FAM172A mRNA level in 16 PC tissues was much lower than that in paired adjacent pancreas by qRT-PCR (t=3.661, *P*=0.002) (Fig. 2A). Both FAM172A and E-cad protein and mRNA levels were much higher in AsPC-1 and BxPC-3 cells compared with that in Miapaca-2 and PANC-1 cells (Fig. 2B,C). The close relationship between FAM172A and E-cad (the key EMT marker) in human PC samples and cell lines drove us to further investigate the role of FAM172A in EMT *in vitro*.

### FAM172A silencing regulated EMT markers and ERK/MAPK signaling in PC cells

**Fig. 3.**
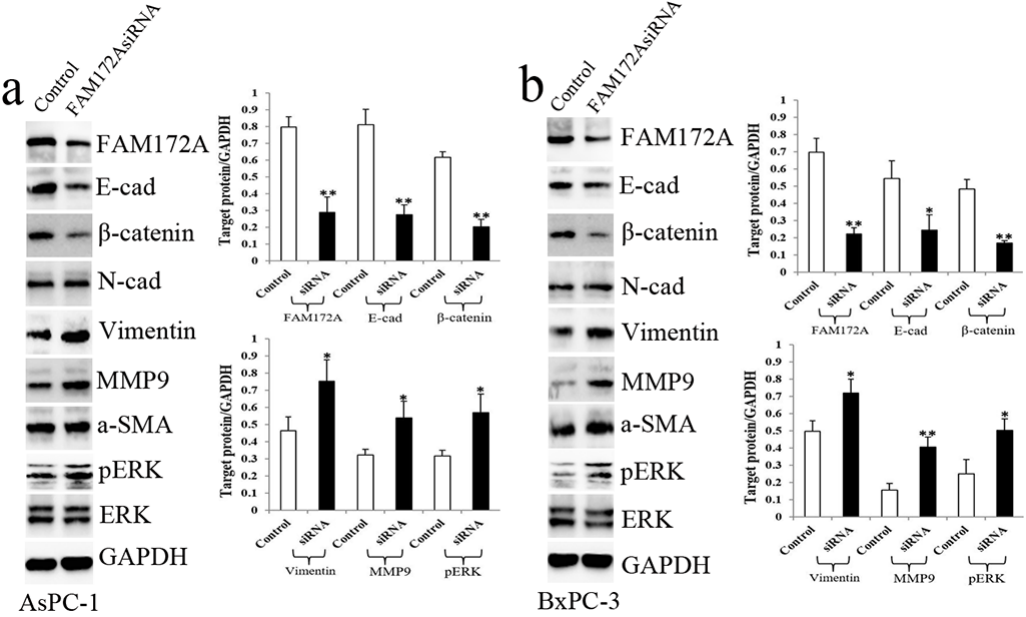
**FAM172A silencing regulated EMT and ERK/MAPK signaling.** (A,B) The protein expression of E-cad, β-catenin, N-cad, Vimentin, MMP9, a-SMA and pERK in AsPC-1 (A) and BxPC-3 cells (B) transfected with FAM172siRNA and siRNAcontrol. White bars, siRNAcontrol group; black bars, FAM172AsiRNA group. E-cad, E- cadherin; N-cad, N-cadherin. Bars indicate±s.e.*P<0.05, **P<0.01 compared with the control.

AsPC-1 and BxPC-3 cells with high FAM172A expression were used for FAM172A silencing experiments. FAM172A protein expression in the FAM172AsiRNA group was much lower than that in the siRNAcontrol group in both cell lines (Fig. 3). FAM172A silencing downregulated EMT epithelial markers E-cad and β-catenin and upregulated mesenchymal markers Vimentin and MMP9, but had no effect in N-cad and a-SMA expression (Fig. 3). Meanwhile, FAM172A silencing upregulated pERK expression in both cell lines (Fig. 3), which indicated that ERK/MAPK signaling might regulate FAM172A-mediated EMT *in vitro*.

### PD98059 inhibited FAM172A silencing-enhanced EMT in PC cells

**Fig. 4.**
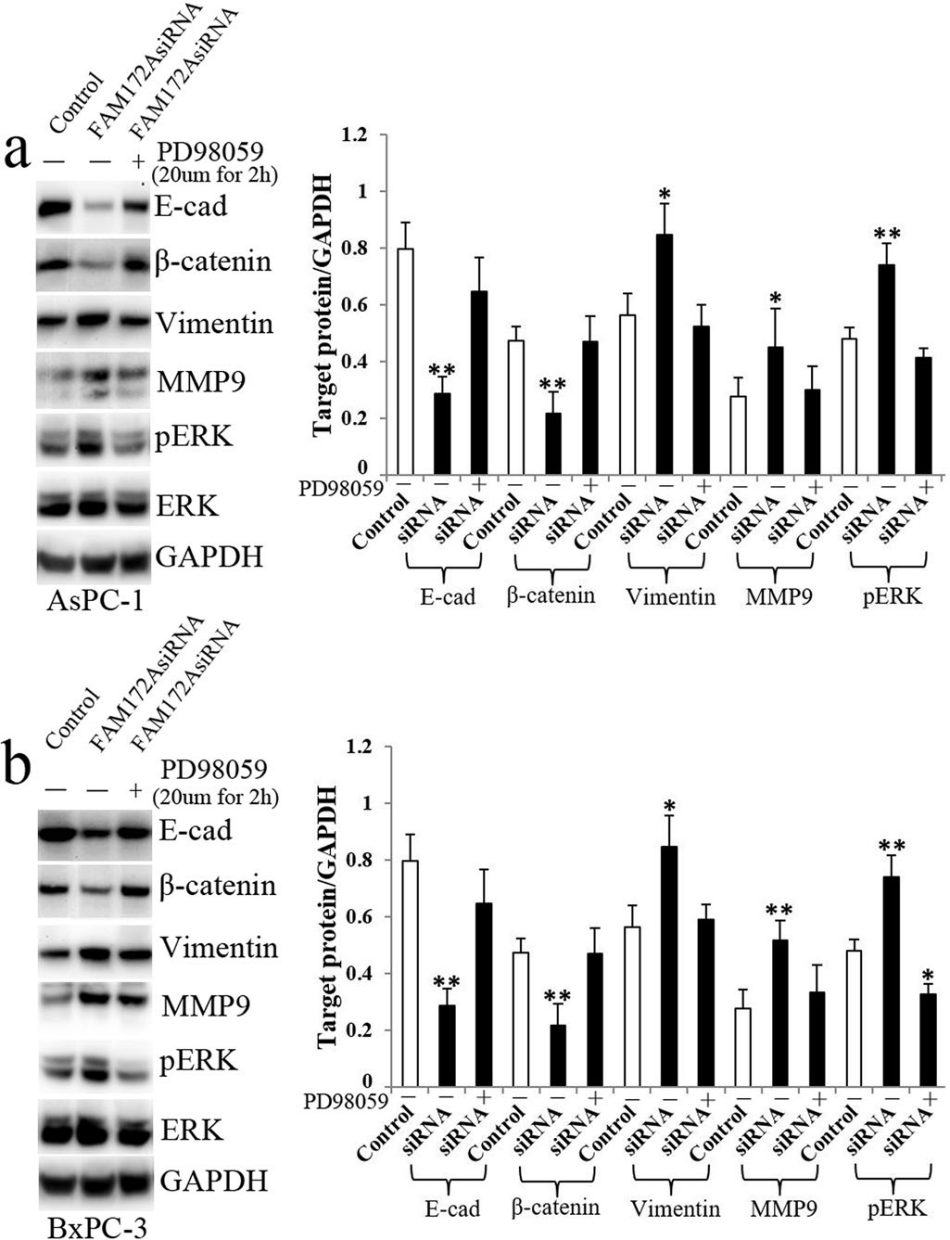
**PD98059 inhibited FAM172A silencing-induced the change of EMT markers.** (A,B) The protein expression of E-cad, β-catenin, Vimentin, MMP9 and pERK in FAM172siRNA and siRNAcontrol transfectedAsPC-1 (A) and BxPC-3 cells (B) with or without PD98059 treatment. White bars, siRNAcontrol group; black bars, FAM172AsiRNA group with or without PD98059 treatment. E-cad, E- cadherin; N-cad, N-cadherin. Bars indicate±s.e. *P<0.05, **P<0.01 compared with the control.

PD98059 was identified as a highly selective inhibitor of MEK1 activation and the MAP kinase cascade (Crews et al., 1992; Cowley et al., 1994). PD98059 binds to the inactive forms of MEK1 and prevents activation by upstream activators (Rosen et al., 1994). First, we found that FAM172A silencing-induced upregulation of pERK that was significantly inhibited by PD98059 (Fig. 4). Meanwhile, PD98059 inhibited FAM172A silencing-induced downregulation of E-cad and β-catenin and upregulation of Vimentin and MMP9 in both AsPC-1 and BxPC-3 cells (Fig. 4).

**Fig. 5.**
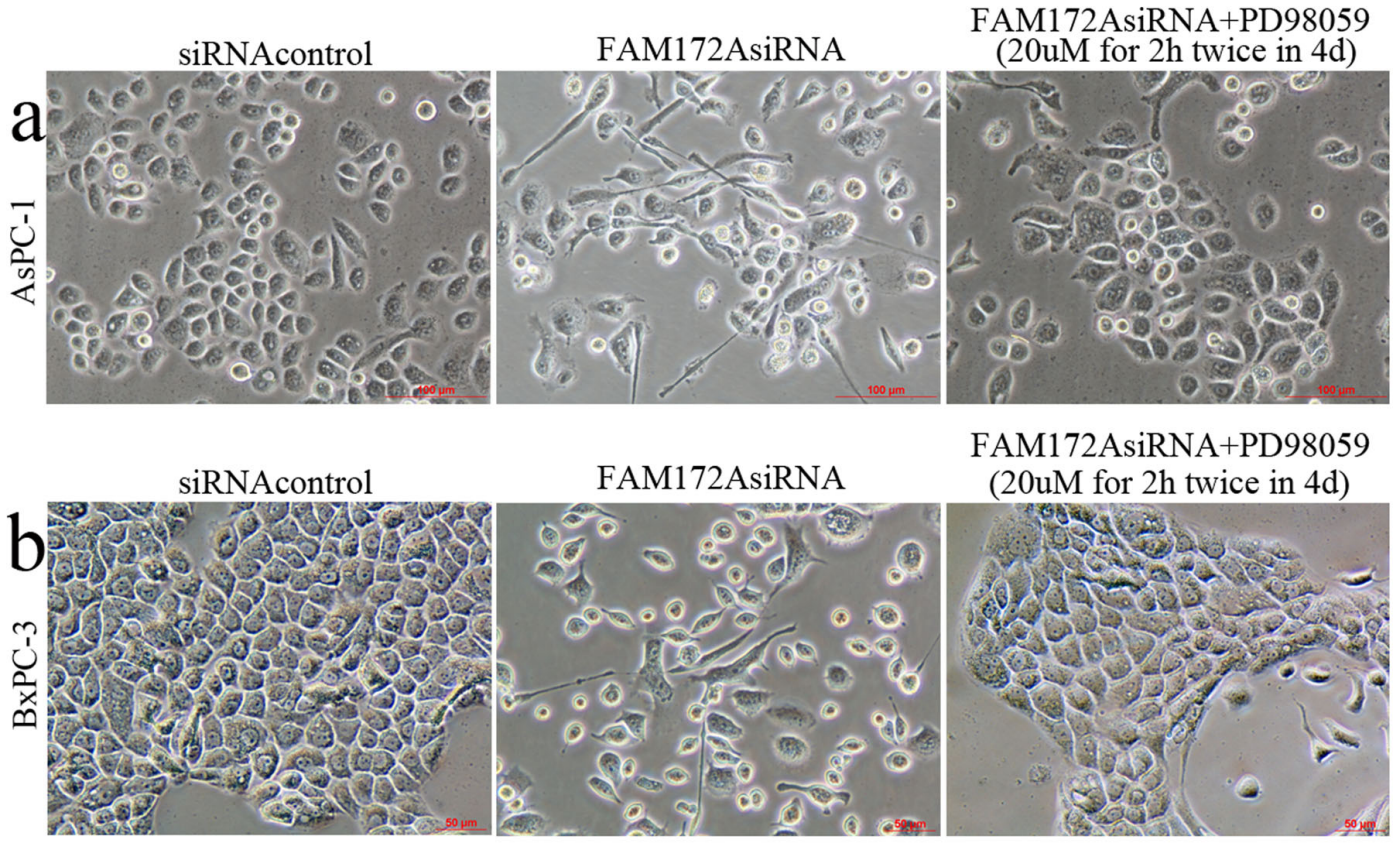
**Cell morphology (×200 magnification) in siRNAcontrol, FAM172AsiRNA and FAM172AsiRNA plus PD98059 groups in AsPC-1 (A) and BxPC-3 cells (B).** Scale bars: A: 100 μm, B: 50 μm.

In addition, FAM172AsiRNA transfected AsPC-1 and BxPC-3 cells presented EMT-like cell morphology: most cells lost their epithelial properties of tight junction, and presented a spindle-shaped and fibroblast-like morphology (Fig. 5). However, PD98059 restored FAM172A silencing-induced EMT-like cell morphology along with the little spindle-shaped and fibroblast-like morphology compared with FAM172AsiRNA groups (Fig. 5). It indicated that FAM172A silencing-induced EMT-like cell morphology in PC cells, which was mediated by ERK/MAPK signaling.

**Fig. 6.**
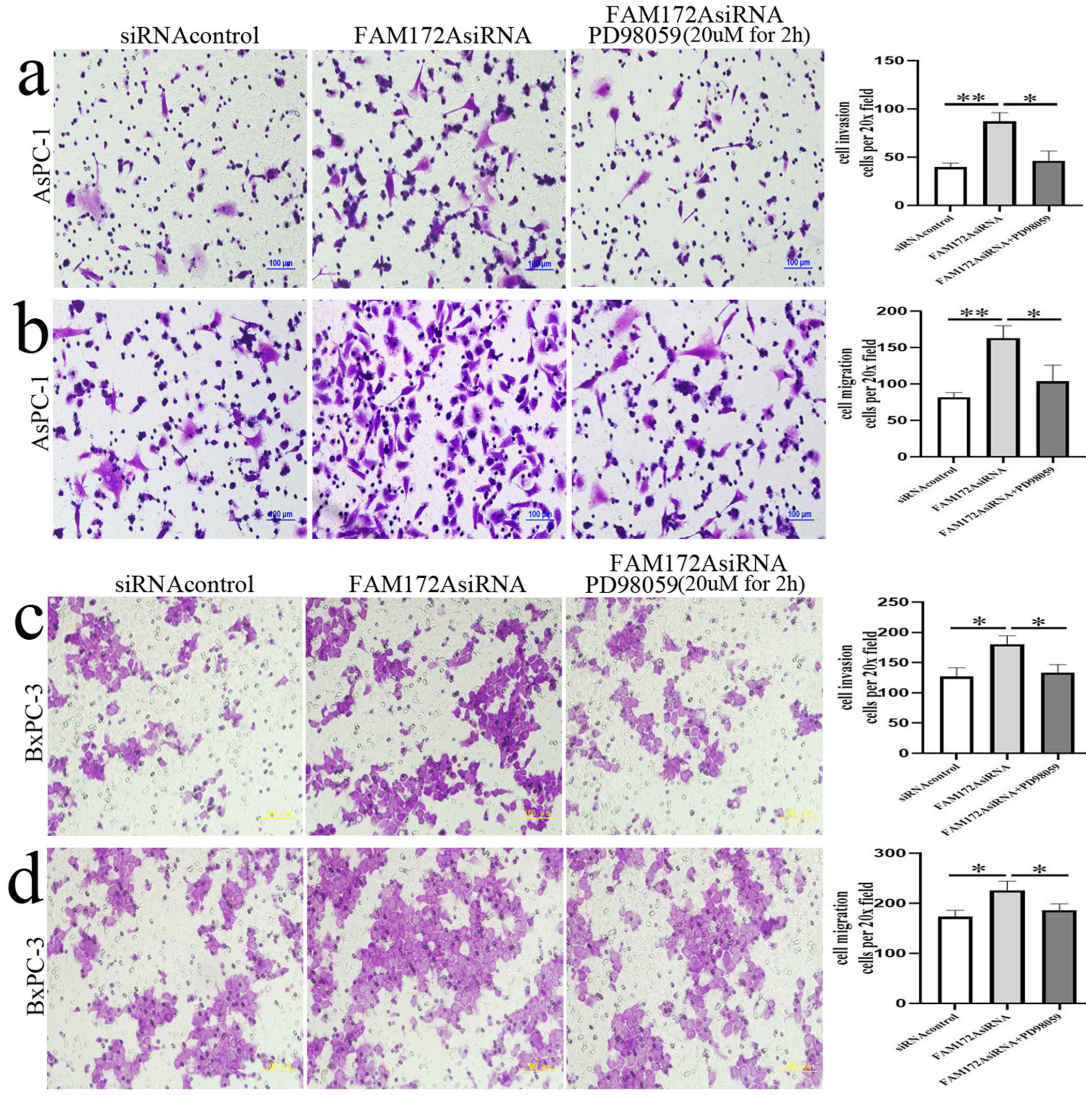
**PD98059 inhibited FAM172A silencing-enhanced cell invasion and migration in PC cells.** (A,B) Cell invasion (A) and migration (B) in siRNAcontrol, FAM172AsiRNA and FAM172AsiRNA plus PD98059 groups in AsPC-1 cells. (C,D) Cell invasion (C) and migration (D) in siRNAcontrol, FAM172AsiRNA and FAM172AsiRNA plus PD98059 groups in BxPC-3 cells. Bars indicate±s.e.*P<0.05, **P<0.01 compared with the control (×200 magnification). Scale bars: 100 μm.

Cell invasiveness in PC is significantly driven by EMT (Wang et al., 2017). In the current study, cell invasion and migration were significantly enhanced in the FAM172AsiRNA group compared with the siRNAcontrol group in AsPC-1 and BxPC-3 cells. However, PD98059 inhibited FAM172A silencing-enhanced cell invasion and migration *in vitro* (Fig. 6). It indicated that FAM172A silencing-enhanced cell invasion and migration in PC cells via regulating ERK/MAPK signaling.

## DISCUSSION

FAM172A, as a newly discovered gene, is little known in the development of cancers. Its function was only reported in liver, colorectal and papillary thyroid cancers (Qian et al., 2016; Feng et al., 2013; Li et al., 2016). However, whether it is an oncogene or a tumor suppressor remains controversial. For example, FAM172A suppresses proliferation and invasion and promotes apoptosis and differentiation in colorectal cancer cells (Qian et al., 2016; Cui et al., 2016), but promotes the proliferation of human papillary thyroid carcinoma cells (Li et al., 2016). In the current study, FAM172A, as a tumor suppressor, inhibits EMT in PC cells via ERK-MAPK signaling, which has not been reported, to our knowledge.

We first found that FAM172A was downregulated in PC tissues and was negatively associated with tumor size, T stage, lymph node metastasis and the poor prognosis of PC patients. FAM172A was also downregulated in liver and colorectal cancer (Qian et al., 2016; Cui et al., 2016). However, FAM172A was upregulated in papillary thyroid cancer (Sheng et al. 2017b). Meanwhile, FAM172A was positively associated with TNM stage, CEA and CA19-9, lymph node involvement and poor prognosis in colorectal cancer (Liu et al., 2017a,b). It indicates that FAM172A exhibits different functions based on different cancer types. Interestingly, we found a positive relationship between FAM172A and E-cad expression in PC tissues and cell lines. Meanwhile, cooperative expression of these two proteins contributes to the better prognosis of PC patients. It is well known that E-cad decrease/loss is a hallmark of EMT in various cancers. For example, miR-151a induces partial EMT by regulating E-cad in non-small cell lung cancer cells (Daugaard et al., 2017). BCL6 induces EMT by promoting the ZEB1-mediated transcription repression of E-cad in breast cancer cells (Liu et al., 2015). Gli1 promotes TGFβ1 and EGF induced EMT in PC cells via downregulating E-cad (Yu et al., 2015). TRIM66 promotes malignant progression of liver cancer by inhibiting E-cad expression through the EMT pathway (Zhang et al., 2019). Therefore, we next investigated if FAM172A regulated EMT in PC cells.

FAM172A silencing significantly induced EMT in both AsPC-1 and BxPC-3 cells, including inducing the increase of Vimentin, MMP9 and the decrease of E-cad and β-catenin expression, stimulating EMT-like cell morphology and enhancing cell invasion and migration. It is well known that ERK/MAPK signaling play a significant role in regulating the initiation of EMT in mammary epithelial and cancer cells. For example, activation of the ERK pathway is required for TGFβ1-induced EMT in normal murine mammary gland epithelial cells (Xie et al., 2004). ERK signaling modulates epigenome to drive epithelial to mesenchymal transition (Navandar et al., 2017). TFF3 contributes to EMT in papillary thyroid carcinoma cells via the MAPK/ERK signaling (Lin et al., 2018). Downregulation of RNF138 inhibits EMT in glioma cells via suppression of the ERK/MAPK signaling (Wu et al., 2018). Calreticulin promotes EGF-induced EMT in PC cells via Integrin/EGFR-ERK/MAPK signaling pathway (Sheng et al. 2017b). In the current study, we first found that FAM172A silencing downregulated pERK expression *in vitro*. Moreover, FAM172A silencing-induced change of EMT classic markers (Vimentin, MMP9, E-cad and β-catenin) was significantly reversed by MEK1 inhibitor PD98059. PD98059 also inhibited FAM172A silencing-induced EMT like cell morphology and cell invasiveness. Previous studies have shown that FAM172A promotes the proliferation of papillary thyroid carcinoma cells via p38/MAPK signaling (2013; Li et al., 2016). FAM172A modulates apoptosis and proliferation of colon cancer cells via STAT1 binding to its promoter (Qian et al., 2016). miR-27a promotes proliferation, migration and invasion of colorectal cancer by targeting FAM172A (Liu et al., 2017a,b). Our study describes a novel signaling pathway involving FAM172A regulating EMT in PC cells via ERK/MAPK signaling, which has not been reported in previous studies.

The limitation in current study is that we do not conduct the transplantation tumor experiment *in vivo*, which would provide strong evidence to support our current conclusion. Second, we have found the potential relationship of FAM172A and ERK/MAPK signaling in mediating the initiation of EMT *in vitro*. However, the detailed molecular mechanism has not been clarified. It is well known that c-Myc, as the major downstream target of ERK/MAPK pathway, is indispensable in EMT development. For example, both c-Myc and MEK1-induced ERK2 nucleus localization are required for TGF-β-induced EMT in prostate cancer (Amatangelo et al., 2012). c-Myc mediates cancer stem-like cells and EMT in triple negative breast cancers cells (Yin et al., 2017). Therefore, whether FAM172A has a specific regulatory effect on c-Myc will be investigated in our future EMT study.

In conclusion, we first found downregulation of FAM172A in PC tissues is negatively associated with advanced clinical significance and poor prognosis of PC patients. FAM172A inhibits EMT in PC via ERK-MAPK signaling, which is identified as a potential gene therapy target in PC.

## MATERIALS AND METHODS

### Tissue samples and cell lines

This study was approved by the academic review board from Huludao Central Hospital. Written informed consent was obtained from each patient. The study methodologies were approved by Huludao Central Hospital and all experiments conform to the Declaration of Helsinki. 65 formalin-fixed PC and paired normal pancreas were collected from postoperative patients who approved and signed consent forms at our hospital from 2005 to 2015. All PC samples were pathologically diagnosed as ductal adenocarcinoma. Additionally, 16 fresh PC tissue samples were randomly selected for late qRT-PCR assays. Human Miapaca-2, PANC-1, AsPC-1 and BxPC-3 PC cell lines were obtained from the cell bank culture collection of the Chinese Academy of Sciences (Shanghai, China), which were cultured in the recommended growth media with 10% FBS (Gibco Invitrogen, Carlsbad, CA, USA).

### ICH assays

IHC was performed as described previously (Sheng et al., 2017a,b). Briefly, 4-µm sections were deparaffinized, dehydrated in ethanol, covered with 0.3% peroxyacetic acid, subjected to antigen retrieval and blocked with goat serum. Then sections were incubated with anti-FAM172A (Abcam, Cambridge, UK, ab121364) and E-cadherin (E-cad, Abcam, ab40772) overnight at 4°C. After washing three times with PBS, sections were incubated with the secondary antibody, treated with streptavidin–peroxidase reagent, visualized with DAB, stained with Hematoxylin and finally detected under microscope. Staining intensity was scored as 0/negative, 1/weak, 2/medium, and 3/strong. Staining range was scored as 0/(<5%), 1/(5–25%), 2/(26–50%), 3/(51–75%), and 4/(76–100%) according to the whole carcinoma. The intensity and extent scores were added together. Tumors with a final score ≥3 were considered to be FAM172A positive and E-cad normal expression.

### Western blot

As described previously (Liu et al. 2017 a,b), whole-cell lysates of PC cells were put into 12% SDS-polyacrylamide gels, transferred to PVDF membrane (Bio-Rad, CA, USA) and incubated with primary FAM172A (Abcam, ab121364), E-cadherin (Abcam, ab40772), β-catenin (Proteintech, Chicago, IL, USA, 51067-2-AP), N-cadherin (N-cad, Abcam, ab98952), Vimentin (Proteintech, 10366-1-AP), MMP9 (Abcam, ab119906), and α-Smooth muscle actin (a-SMA, Abcam, ab7817), pERK (Cell Signaling Technology, Beverly, MA, USA, #9101), ERK (Cell Signaling Technology, #4695) and GAPDH (Proteintech, 60004-1-Ig) antibodies overnight. Then membranes were incubated with secondary antibodies (Proteintech) at room temperature and were visualized with the ECL machine (Thermo Biotech Inc., USA). PC cells were pretreated with 20 µM of MEK1 inhibitor PD98059 (Cell Signaling Technology) for 2 h before western blotting. The experiment was repeated three times.

### Real-time quantitative PC

As described previously (Fan et al., 2019), qRT-PCR was analyzed in a Light Cycler 2.0 with the Light Cycler kit (Takara Bio, Otsu, Japan) for the following conditions: 95°C for 30 s, 40 cycles of 95°C for 5 s, and 60°C for 30 s. The primers: FAM172A, 5′-CGACTGGCGAACTGGAAG -3′ and 5′-GAGCTCAAGGAAATAGACATCAATC -3′; E-cad, 5′-CAGCGTGTGTGACTGTGAAG -3′ and 5′-AAACAGCAAGAGCAGCAGAA -3′. GADPH, 5′-CATGAGAAGTATGACAACAGCCT -3′ and 5′-AGTCCTTCCACGATACCAAAGT -3′. Quality of the PCR products was monitored with post-PCR melt-curve analysis using the ΔΔCt calculation.

### RNA interference

The FAM172AsiRNA sequences were: sense: 5′-GCCACTGAGAGTGAACCAAAG -3′, which were synthesized from GenePharma company (Shanghai, China). siRNA transfections (20 µM) were mixed with Oligofectamine 3000 (Invitrogen, Carlsbad, CA, USA) for transient transfection following the manufacturer’s instructions.

### EMT construction

As described previously (Fan et al., 2019), FAM172AsiRNA and siRNAcontrol transfected AsPC-1 and BxPC-3 cells were cultured with growth media containing 1% fetal calf serum (FBS) twice within 4 days to better induce EMT. The change of EMT-like cell morphology, the protein level of EMT markers and cell mobility were used for detecting the EMT construction. For EMT-like cell morphology in siRNAcontrol, FAM172AsiRNA and FAM172AsiRNA plus PD98059 groups, we pretreated PC cells with PD98059 (20 µM) for 2 h twice within 4 days. DMSO, as the dilution of PD98059, was used as the control.

### Cell invasion and migration assays

Briefly, FAM172AsiRNA and siRNAcontrol transfected AsPC-1 and BxPC-3 cells (pretreated with 20 µM of PD98059 for 2 h only once) was seeded onto 8.0-µM pore size membrane inserts (Corning Inc., NY, USA) coated with matrigel (BD Biosciences, Sparks, MD, USA) in 24-well plates with FBS-free growth media. Growth media plus 10% FBS was added to the bottom wells as a chemoattractant. 24 h later, cells that moved to the underside of the inserts were stained with Crystal Violet Hydrate (Sigma-Aldrich, St Louis, MO, USA). The migratory cells were counted in five random fields per well. Results were expressed as cells migrated per field and repeated three times. The transwell assay was performed in a similar way without matrigel.

### Statistical analysis

Under SPSS software 20.0 (SPSS, Chicago, IL, USA), paired nonparametric test, chi-squared test and spearman correlation test were used for IHC assays, respectively. The log-rank test and Cox's regression was used to evaluate the postoperative survival time of PC patients. Western blotting, qRT-PCR and transwell assays *in vitro* were described as means±s.e. (standard deviation) and were compared via *t*-test. *P*-value is presented as follow: *P<0.05; **P<0.01.
